# Docking and molecular dynamics simulations of the ternary complex nisin_**2**_:lipid II

**DOI:** 10.1038/srep21185

**Published:** 2016-02-18

**Authors:** Sam Mulholland, Eleanor R. Turpin, Boyan B. Bonev, Jonathan D. Hirst

**Affiliations:** 1 School of Chemistry, University of Nottingham, University Park, Nottingham NG7 2RD, UK; 2 School of Pharmacy, University of Nottingham, Queen’s Medical Centre, Nottingham NG7 2RD, UK; 3 School of Life Sciences, University of Nottingham, Queen’s Medical Centre, Nottingham NG7 2UH, UK

## Abstract

Lanthionine antibiotics are an important class of naturally-occurring antimicrobial peptides. The best-known, nisin, is a commercial food preservative. However, structural and mechanistic details on nisin-lipid II membrane complexes are currently lacking. Recently, we have developed empirical force-field parameters to model lantibiotics. Docking and molecular dynamics (MD) simulations have been used to study the nisin_2_:lipid II complex in bacterial membranes, which has been put forward as the building block of nisin/lipid II binary membrane pores. An Ile1Trp mutation of the N-terminus of nisin has been modelled and docked onto lipid II models; the computed binding affinity increased compared to wild-type. Wild-type nisin was also docked onto three different lipid II structures and a stable 2:1 nisin:lipid II complex formed. This complex was inserted into a membrane. Six independent MD simulations revealed key interactions in the complex, specifically the N-terminal engagement of nisin with lipid II at the pyrophosphate and C-terminus of the pentapeptide chain. Nisin^2^ inserts into the membrane and we propose this as the first step in pore formation, mediated by the nisin N-terminus–lipid II pentapeptide hydrogen bond. The lipid II undecaprenyl chain adopted different conformations in the presence of nisin, which may also have implications for pore formation.

Resistance to antibiotics and the spread of infectious diseases in a globalised society present significant challenges to infection management and disease control. In addition, control of food spoilage bacteria is an essential component of securing a nutritious and safe food supply. The development of new antibacterial compounds, which could lead to anti-spoilage food packaging, was also one of the strategies identified by the World Health Organisation to combat antibiotic resistance, where antibiotic peptides have been recognised as one of six promising areas of research^1^. Peptidoglycan-targeting antimicrobial peptides are presently under-utilized in bacterial management and resistance remains uncommon. Used alone, or as facilitators in combinations with other antimicrobials, these compounds hold much promise and may offer a route to novel strategies for bacterial control^2^.

Lanthionine antibiotics (lantibiotics) are a group of antimicrobial peptides that have high efficacy against Gram-positive bacteria, good stability and low toxicity to humans^3^,^4^,^5^. Lantibiotics contain unusual dehydrated residues, dehydroalanine (Dha) and dehydrobutyrine (Dhb), derived from post-translation, enzyme-mediated dehydration of serine and threonine residues. Another unique feature is the presence of macrocyclic rings joined by thioether bonds, made by fusing a cysteine thiol to Dha or Dhb, forming either a lanthionine ring or β-methyl-lanthionine ring, respectively^6^.

A commercially developed and well-characterised lantibiotic, nisin (Fig. 1a), is produced by the Gram-positive bacterium *Lactococcus lactis* and is effective against *Staphylococci, Clostridia* and *Lysteria*^7^. Currently having numerous applications in food protection worldwide, assigned the E number E234, nisin is a non-toxic 34 amino acid peptide, which is conformationally stabilised by five thioether macrocyclic rings. It contains three dehydrated residues, of which Dha5 is essential for spore outgrowth inhibition^8^.

The peptide is positively charged and readily binds negative and neutral membranes^9^. The antimicrobial action of nisin arises from molecular recognition of lipid II^10^ (Fig. 1b), a specific surface receptor on bacterial plasma membranes. Lipid II is the mature intermediate in peptidoglycan synthesis and the target for lanthionine antibiotics from all classes, as well as glycopeptide antibiotics, such as vancomycin^11^. The molecular architecture of lipid II is N-acetylmuramate (MurNAc) with a muropeptide at position 3, 4–1′, N-acetylglucosamine (GlcNAc) and 1-pyrophosphate-linked undecaprenyl chain anchored in the bacterial membrane.

Nisin exhibits multiple modes of antimicrobial action. On the molecular level nisin disrupts membranes containing lipid II by binding to the receptor with a 2:1 nisin:lipid II stoichiometry^12^. It has been proposed that nisin_2_:lipid II ternary complexes subsequently assemble into higher-order multimeric pores, which dissipate transmembrane potentials and solute gradients^13^. At the mesoscopic scale, nisin deregulates bacterial division and cell wall morphogenesis^14^ and inhibits spore outgrowth^8^,^15^. Nisin binds to lipid I (11PP-MurNAc-5Pep) as strongly as it does to lipid II^16^.

NMR studies of nisin in solution^17^,^18^,^19^,^20^ and membrane-mimetic detergent micelles^21^ have shown that rings A, B and C are joined to the inter-linking rings D and E by a flexible region. The linear tails at the N- and C-termini are also flexible^22^. An NMR study of 1:1 complexes of a nisin with a truncated lipid II-variant in DMSO indicates engagement by rings A and B of nisin with the pyrophosphate group in lipid II^23^. In addition, solid state NMR has revealed a key role of the N-terminus in target engagement^10^. We have previously studied analogs of the target-recognition motif comprising residues 1–12 of nisin by molecular dynamics (MD) simulations. Disulfide-linked macrocyclic peptide analogs of nisin containing rings A and B were found to be similar in conformation to the natural peptide, but could form in the absence of the nisB/C enzymatic machinery^24^.

Some previous simulation work has been completed on lipid-Il and antimicrobials within a model bacterial membrane environment. Jia and co-workers^25^ have modeled the recognition of lipid-II by vancomycin, and then the dimerization of these complexes, using the GROMOS 54A7 force field. Chugunov *et al.*^26^ have made an extensive analysis of the behavior of lipid-II in a membrane using MD simulations, identifying distinct conformations of the lipid-II tail in the bilayer and an induced amphiphilic ‘landing terrain’ pattern on a model bacterial membrane surface that is not seen in a reference membrane. More recently we have developed CHARMM empirical force-field parameters for the unusual dehydrated amino acids present in lantibiotics^27^. MD simulations of nisin in an aqueous environment interacting with a phospholipid bilayer were conducted and the conformation and dynamics of the peptide agreed with solid state and solution NMR^20^. Koch and co-workers^28^ published the first computational study of a nisin-lipid II complex, by inserting the NMR structure (PDB code 1WCO) into a membrane, where they found the presence of nisin reduced the mobility of lipid II; however, beyond a deeper insertion of the tip of the lipid II tail they did not see any evidence of pore formation with the 1:1 ratio. Building on these previous studies, we focus for the first time on the biologically relevant ternary nisin_2_:lipid II complex, using docking calculations and MD simulations to investigate the mechanism of nisin binding and targeting of lipid II in membranes.

Molecular docking offers a starting model of the molecular interactions between ligand and receptor and provides a rapid means for generating hypotheses of the mode of recognition and molecular interactions. MD simulations are subsequently used to incorporate conformational flexibility into both partners of the complex and permit us to assess whether key interactions persist, in the context of the full atomistic molecular mechanics force-field. As yet, the docking site for a second nisin molecule on lipid II has not been identified and reported in the literature, nor has the mechanism of pore-formation been described. The present work proposes a binding site at the C-terminus of D-Ala5 of lipid II and demonstrates that this 2:1 complex is stable in MD simulations. The second nisin is inserted into the membrane in three of the six trajectory runs and we propose that this is the first step of pore formation. The N-terminal engagement of nisin with lipid II has been explored further by considering a single point mutant, Ile1Trp, inspired by subtilin and allowing membrane anchoring of the peptide^29^, modelled *in silico* and its binding affinity to lipid II has been computed. Nisin models have also been docked to lipid I to explore conformational differences compared with nisin-lipid II complexes.

## Methods

### Molecular dynamics simulation of lipid II and nisin-lipid II complexes

MD simulations of a single lipid-II molecule in a membrane were conducted to generate starting conformers for docking and to compare with the simulations of nisin_2_-lipid II complexes. Simulations were performed with the CHARMM 36 force-field^30^. The building of the molecular system was performed using CHARMM. The membrane was set up following the protocol described in^27^: using the CHARMM-GUI^31^,^32^ membrane builder^33^,^34^, a solvated lipid bilayer was generated with a composition of 3:1 1-palmitoyl-2-oleoyl-*sn*-glycero-3-glycerol (POPG): 1-palmitoyl-2-oleoyl-*sn*-glycero-3-phosphoethanolamine (POPE) lipids, to represent a Gram-positive bacterial membrane^26^. Each leaflet of the membrane contained 225 POPG lipids and 75 POPE lipids and the membrane surface area was 135 × 135 Å^2^ in the x-y plane. This choice of system size is based on similar simulation studies of lipid II inserted in a bilayer^26^ and the interaction between vancomycin and lipid II^25^. The depth of the water solvent layer above and below the bilayer was 40 Å and contained 442 neutralizing potassium ions. Initial coordinates for lipid II were taken from the first conformer in PDB entry 1WCO^23^, with the nisin molecule deleted. The tail of lipid II was extended to 11 isoprenyl units to match the wild-type. The lipid II molecule was orientated so that the tail was aligned parallel with the membrane in the z-axis and inserted into the membrane with the head group at the same height as the membrane-water interface. Any water molecules, and the three or four POPG or POPE molecules where the head groups overlapped with the lipid II head groups, were deleted.

All minimization, equilibration, and production dynamics were performed using NAMD^35^ with a time step of 2 fs. Periodic boundary conditions were applied using a cuboid cell with starting dimensions of 135 Å × 135 Å × 100 Å. The SHAKE algorithm^36^ was applied to all bonds to hydrogen atoms. The non-bonded cut-off was 12 Å and the non-bonded neighbor list was updated at every time step. Long-range electrostatics were treated using the particle-mesh Ewald method^37^. Initial minimization was performed using the standard NAMD minimization algorithm, which is a combination of conjugate gradient and line search methods. The minimization was performed in stages of 300 steps with all atoms, except the lipid and lipid II tails, which were held fixed; 300 steps with the lipid II molecule fixed and finally 300 steps where all atoms were allowed to relax. This was followed by 1000 steps of minimization and the first 0.5 ns phase of equilibration at 300 K where the lipid tails are relaxed and all other atoms are fixed. In the second equilibration phase, 2500 steps of minimization and a further 0.5 ns of equilibration were performed with the lipid II molecule fixed. In the next equilibration stage, all the atoms were relaxed for 2500 steps of minimization, followed by 10 ns of equilibration in the NPAT ensemble where the surface area in the *x-y* plane was also kept constant to maintain a biologically relevant lipid density^38^. Production dynamics were run at 300 K in the NPT ensemble.

The nisin_2_:lipid II complexes formed from docking calculations (described in the next section) were simulated in a hydrated lipid bilayer using the same parameters as above, unless described otherwise. The coordinates for the nisin_2_-lipid II complex were taken from the docking calculations with the 11 prenyl units of the tail of lipid II rebuilt in an extended conformation using CHARMM. Coordinates for the C-terminus of the nisin molecules were taken from the first conformer in 1WCO and rebuilt into the nisin_2_:lipid II following alignment of the first 12 nisin residues. The lipid II was orientated so that the principal geometric axis of the tail was parallel to the membrane along the z-axis and inserted into the membrane with the head group at the same height as the membrane-water interface. Three POPG and POPE lipid head groups that overlapped with the lipid II head group were deleted; and the corresponding number removed from across the opposite leaflet to maintain comparable surface areas. Any water molecules that overlapped with the nisin molecule or lipid II were deleted. The minimization was performed in stages of 1000 steps with all atoms except the lipid and lipid II tails held fixed; 1000 steps with the nisin_2_:lipid II complex fixed; 1000 steps with the nisin molecules fixed and finally 1000 steps where all atoms were allowed to relax. This was followed by 2500 steps of minimization and the first 0.5 ns phase of equilibration at 300 K where the lipid tails are relaxed and all other atoms are fixed. In the second equilibration phase 2500 steps of minimization and a further 0.5 ns of equilibration were performed with the nisin molecules fixed. Post-production processing and analysis was performed using the correl, corman and hbond modules in CHARMM, with the entirety of the production trajectories analysed unless otherwise stated; for the radial distribution function all six were trajectories were treated together as an ensemble.

Three independent simulations of 100 ns were conducted of lipid-II in a membrane and 3 × 90 ns and 3× 100 ns simulations of nisin_2_:lipid II in a membrane (570 ns production dynamics sampling in total). The thermodynamic stability of the MD simulations was confirmed by examining the kinetic energy, potential energy, total energy, temperature and pressure (data not shown). The conformational preferences of the lipid II tail were characterised by aligning the lipid II head group from each trajectory frame and then clustering by the dihedral angle of the single bonds in the polyprenyl chain, using the cluster command. The average mass weighted RMSD of the lipid-II head group after alignment was 1.91 Å.

### Docking calculations

To generate a starting structure of the nisin_2_:lipid II complex we docked consecutive nisin molecules to lipid-II. All docking calculations were performed using AutoDockVina^39^. Initially, in order to confirm the suitability of the AutoDockVina scoring function and parameter selection in this study, re-docking was performed of the peptide and lipid-II using the coordinates from the NMR structure of a single nisin molecule in complex with isopentenyl lipid II in DMSO^23^ (conformer 1, PDB entry 1WCO). It was confirmed from these calculations that full 34-residue nisin and a truncated 12-nisin accurately calculate the binding conformation and hydrogen bond interactions identified by Hsu *et al.*^23^ (data not shown).

Three lipid II models were used in our docking calculations: one model is the first experimental structure taken from 1WCO, while the other two are from final trajectory frames from two of the MD simulation of lipid II within a membrane environment described in the previous section. The first model contains a single isoprenyl unit and the others were shortened to three prenyl units for docking. These truncated lipid tails were used, as nisin is not thought to bind to the hydrophobic poly-prenyl chain embedded in the inaccessible membrane and docking calculations are more efficient with fewer atoms.

Lipid II was treated as the receptor and nisin as the ligand. A united atom model was used, in which all non-polar hydrogen atoms were merged using AutoDockTools^40^. Gasteiger-Marsili partial charges^41^,^42^ were assigned to each atom in the nisin peptide. Ten backbone torsional degrees of freedom were specified for nisin: Ile1 *Φ* and *ψ*, Dhb2 *Φ* and *ψ*, D-Ala3 *Φ*, Cys7 *ψ*, D-Abu8 *Φ*, Cys11 *ψ*, Lys12 *Φ* and *ψ*; the macrocyclic rings and C-terminus were treated as rigid. A cubic grid box of side 100 Å was centred on the pyrophosphate moiety of lipid II. Docking was run specifying an exhaustiveness parameter of 200. Increasing the exhaustiveness parameter increases the search time linearly and decreases the probability of not finding the minimum exponentially; for more information please refer to the AutoDockVina paper^39^.

To generate the Ile1Trp nisin mutant, point mutagenesis of the nisin molecule from the first conformer of 1WCO was performed using Discovery Studio 2.0 and minimised using a Dreiding forcefield^43^. The Ile1Trp mutant was docked to the three models of lipid II individually, with all docking parameters as above, including the same ten backbone torsional degrees of freedom as the *wt-*nisin. Coordinates for a lipid I model were generated by removing the GlcNAc moiety, and terminating the oxygen of MurNAc as a hydroxyl group, of the 1WCO lipid II models used above.

## Results and Discussion

### Docking nisin

**Figure 2:**
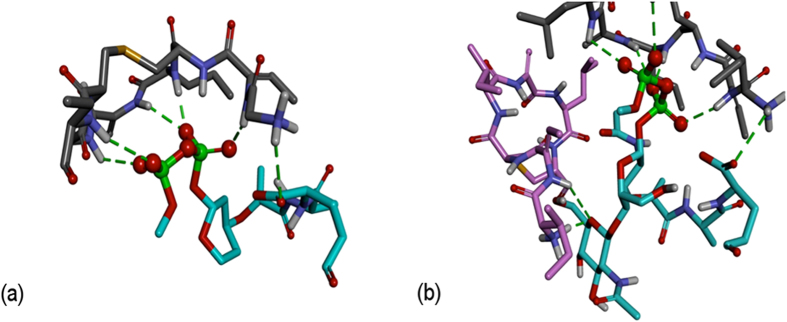
(**a**) The conformation of the first seven residues of the nisin peptide (ring A) (shown in grey carbon atoms) docked on lipid II (shown in cyan carbon atoms) (lipid II modelled after 1WCO [23]). (**b**) Docked structure of nisin^2^ (pink carbon atoms) onto the complex formed between the first nisin molecule (grey carbon atoms) and lipid II (cyan carbon atoms. For clarity, the isoprenyl chain and residues not involved in hydrogen bonding are omitted. Hydrogen bonds are shown in green dashes.

In common with all docking programmes, AutoDock Vina produces a binding affinity that is useful for comparing different conformations and ligands^39^. These binding affinities are not quantitative evaluations of Gibbs free enegies but can still be used to gain insight into the differences between nisin interacting with lipid I and lipid II. The first nisin docked onto the DMSO lipid II model and the lowest energy complex had a calculated binding affinity of −11.1 kcal/mol. The conformation of the first two nisin rings, residues 1–12, was maintained as proposed by Hsu *et al.*^23^ while docking onto rigid lipid II (Fig. 2). After introducing additional flexibility into the pentapeptide chain of lipid II, a new hydrogen bond formed between the N-terminus of nisin and the γ-D-glutamate on lipid II, giving a total of six hydrogen bonds. An attractive electrostatic interaction was also present between the N-terminus and the δ-oxygen of γ-D-Glu2 on lipid II, along with hydrophobic interactions between the sidechain of Ile4 of nisin and a methyl group on the MurNAc sugar. Little is known about the mechanism of initial interaction between nisin and lipid II and these N-terminal interactions, previously reported by us using solid state NMR^10^, are reconciled here with the solution NMR structure in DMSO^23^. The latter reference reports multiple contacts between nisin rings A / B and **P**_2_O_7_ of lipid II but unrestrained N-terminal Ile1, while the former study shows direct dipolar coupling between H**N**-Ile1 and the **P**_2_O_7_ of lipid II. Our model replicates all available NMR constraints, both from solution and solid state NMR studies, and supports the hypothesis^44^ in which N-terminal engagement of the peptide is the first step in target recognition and complex formation.

The equilibrated membrane coordinates of lipid II are also used here in docking calculations to provide a more physiologically relevant structure for the nisin to dock on. Nisin was docked to the three different models of lipid II and the binding affinity is summarised in Table 1. The conformations of nisin after docking to the two full length models of lipid II, obtained from MD simulations, are very similar (Figure S3). Rings A and B are almost identical with only the N-terminus assuming different conformations. The difference in binding affinity can be rationalised by observing that the N-terminus in the higher affinity conformation has additional buried hydrophobic surface area, from the two additional isoprenyl units interacting with the side chain of Leu16.

**Figure 3:**
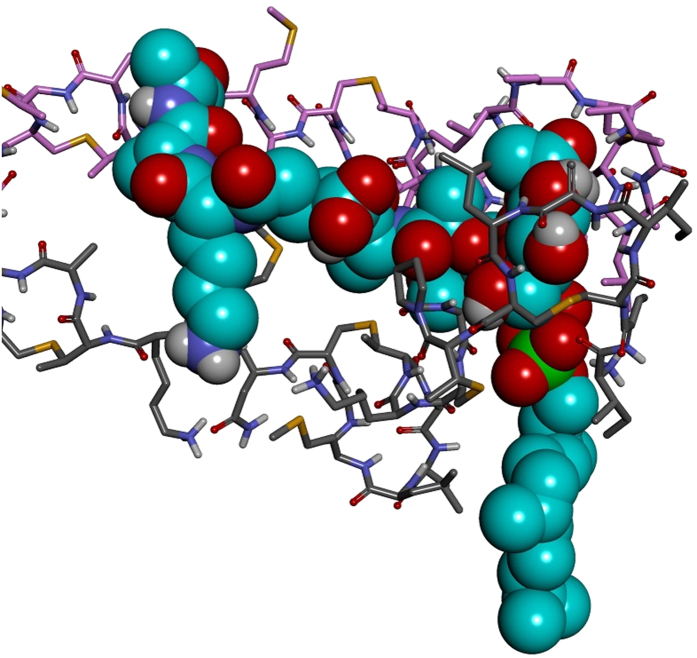
Structure of the complex formed by docking nisin^2^ (pink carbon atoms) onto the 3-isoprenyl-lipid II/nisin complex (first nisin shown in grey carbon atoms and lipid II shown in cyan carbon atoms CPK model).

The lowest energy complex between nisin and DMSO model lipid II, obtained from the first docking stage, was used as a target for docking a second nisin molecule with the aim of obtaining a ternary nisin_2_:lipid II complex. This represents a limitation of the methodology used here, as the lowest energy complex of nisin:lipid II might not correspond to the nisin^1^:lipid II conformation; however, it is assumed that the conformation would be reasonably close given the similarity to 1WCO and that energetic relaxation during the MD stage would move away from metastable states. The calculated binding affinity of nisin^2^:[nisin_1_:lipid II] was −11.7 kcal/mol. The second nisin molecule (nisin^2^) was observed to interact with GlcNAc by forming two hydrogen bonds at the N-terminus (Fig. 2). There was also a hydrophobic interaction between Ile4 nisin^2^ and Leu6 nisin. A second nisin molecule was docked onto the complex of 3-isoprenyl-lipid II and nisin with a calculated binding affinity of −13.6 kcal/mol (Fig. 3). Two hydrogen bonds were observed between D-Ala3 and Ile4 on nisin and GlcNAc on lipid II and the two nisin molecules enrobe the disaccharide of the lipid II.

### 1le1Trp mutant docking

The picture of nisin:lipid II interactions that emerges from our MD simulations presents the opportunity to investigate nisin mutants. Some nisin mutations, created using random mutagenesis, show enhanced activity against Gram-positive bacteria^45^. Of a particular interest is Ile1Trp, as tryptophan has been shown to serve as a membrane protein anchor near the lipid carbonyl region in membranes^29^. In addition, Trp1 occurs naturally in subtilin, a related lantibiotic with comparable or marginally higher activity than nisin^44^. The N-terminus of subtilin plays a crucial role in its activity and deletion of its N-terminal Trp residue abolishes activity altogether^44^. Here, we dock Ile1Trp nisin to our lipid II models to assess any specific target recognition role this mutation may play (Table 1).

Both the DMSO and 1-iso models of lipid II show that Ile1Trp nisin has stronger binding affinity by more than 2 kcal/mol than the wild-type. The 3-isoprenyl model, however, shows only 0.2 kcal/mol difference due to the adoption of a different N-terminal conformation by the Trp. This reinforces the significance of the N-terminal interaction of nisin with lipid II as seen in the MD simulations and in the solid state NMR study. N-terminal Ile1Trp nisin has been proposed to have higher activity than the *wt-*peptide. An *in silico* N-terminal mutation of nisin reveals higher binding affinity than the wild-type on lipid II models.

### *wt-*nisin docking to lipid I

Brötz *et al.*^16^ and Bonev *et al.*^10^ have shown that nisin binds in a very similar way to both lipid I and lipid II. A docking calculation of nisin on lipid I was performed using the final conformations of nisin and lipid II, obtained from the previous docking stage. The docking confirmed the similarity between lipid I and lipid II as nisin targets. The calculated binding affinity was −11.2 kcal/mol and the highest ranking binding mode showed that the pyrophosphate-engaging structure was preserved. The same hydrogen bonding, electrostatic and hydrophobic interactions were present.

A second nisin was then docked onto the nisin:lipid I complex and compared to the one formed when docking a second nisin onto the nisin:lipid II complex and the conformations are very different. The calculated binding affinity when docking to nisin:lipid I is lower, −10.2 kcal/mol, with a contribution from four intermolecular hydrogen bonds (Figure S1) and a complex hydrogen bonding network with MurNAc that is not accessible with lipid II.

### MD simulations of lipid II and nisin_2_:lipid II complex

**Figure 4:**
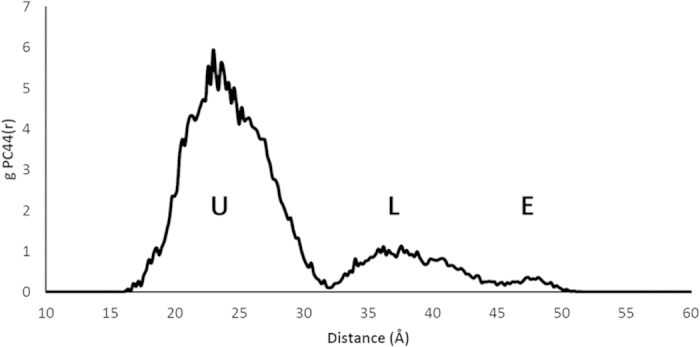
Radial distribution function of the distance between the final carbon atom in the lipid II chain and the pyrophosphate in a hydrated bilayer simulation. U–U-conformation; L–L-conformation; E–extended.

**Figure 5:**
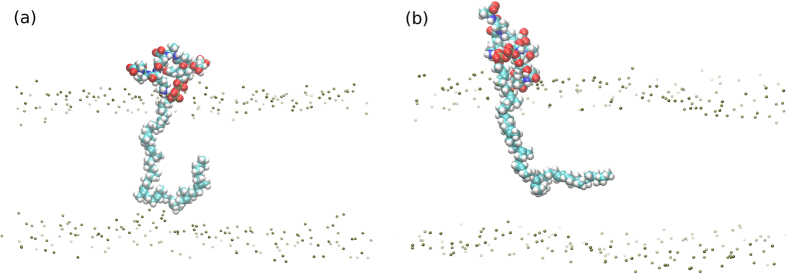
(**a**) U-conformation and (**b**) L-conformation of the lipid II undecaprenyl chain in a lipid bilayer; membrane phosphates are shown to illustrate level of lipid head groups, other atoms removed for clarity.

The first simulation phase was lipid II only, where in agreement with Chugunov *et al.*^26^, the lipid II head group is anchored by the pyrophosphate moiety at the bilayer-water boundary (Figure S2), whilst the tail is free to move within the hydrophobic region of the bilayer (Fig. 4). The U or L conformations are favored, in which the tail end is localized away from the opposite leaflet and moving within the milieu of lipid tails. The U conformation populates the first peak (at ~17–31 Å) in the radial distribution function of the distance between the pyrophosphate centre and the terminal carbon atom in the tail, *g*_*PC44*_(*r*) (Fig. 5a). The L conformations (Fig. 5b) populate the second peak in *g*_*PC44*_(*r*).

**Figure 6:**
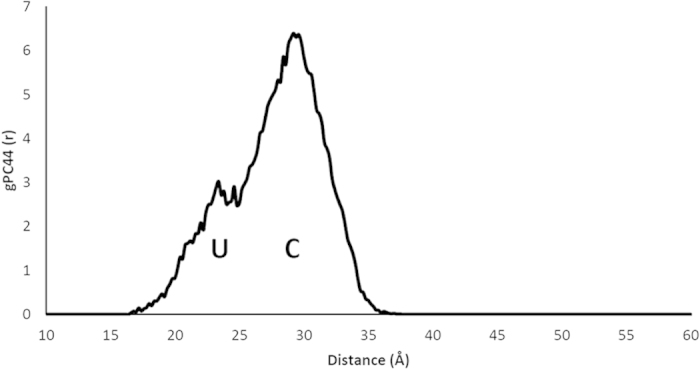
The radial distribution function of the lipid II tail terminus with respect to pyrophosphate in the hydrated bilayer simulation with two nisin molecules bound to the complex. The value of density set is 2×10^−6^, the number density of the single tail carbon atom in a sphere with 50 Å radius.

To investigate the interactions in ternary nisin_2_/lipid II complexes in more detail we carried out six independent MD simulations in a hydrated lipid membrane. The complexes remained parallel to the membrane surface with the pyrophosphate moiety of the lipid II at the edge of the membrane/water interface (Figure S4) and the bound nisin molecules stretching across the surface of the membrane. The radial distribution function *g*_*PC44*_(*r*) of lipid II (Fig. 6) is different in the presence of nisin. The highest frequency population corresponds to a coiled tail (main peak at ~30 Å, Fig. 6). This constricted conformation may play an important role in the assembly of higher-order multimeric complexes during pore formation.

Receptor recognition by nisin is mediated by the formation of multiple hydrogen bonds, which are likely to make an important contribution to the overall stability of the nisin:lipid II membrane complexes. Hydrogen bonds were observed to form between each nisin and all parts of the lipid head group (disaccharide, pentapeptide chain and pyrophosphate). The cut-off distance for defining a hydrogen-acceptor pair was 2.4 Å and there was no cutoff for the bond angle. Structures were sampled every 10 ps. Table 2 reports the percentage of trajectory frames where a hydrogen bond is formed between a polar atom in nisin and a polar atom in either the pyrophosphate group, disaccharide or pentapeptide of the lipid II head group.

**Figure 7:**
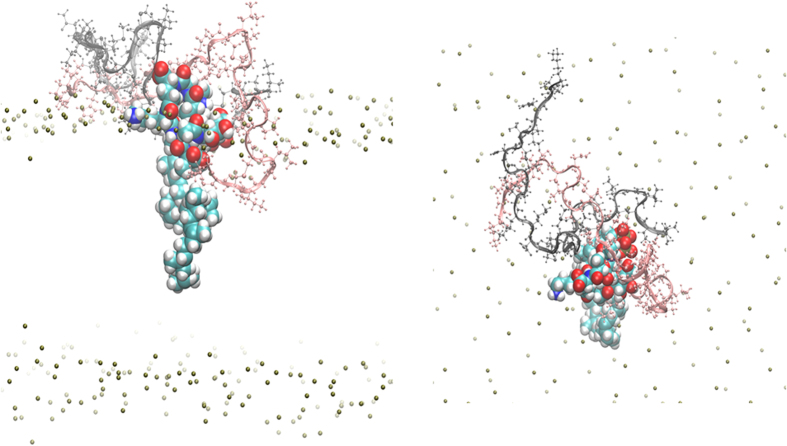
Trajectory snapshot from the MD simulation of the nisin_2_:lipid2 complex in a hydrated lipid membrane. Nisin^1^ is displayed in grey carbon atoms and molecular surface, nisin^2^ in pink carbon atoms and molecular surface, lipid II in cyan carbon atoms. Hydrogen bonds are displayed as green dashes. View of x-y plane.

The simulation revealed N-terminal interaction of Ile1 in nisin^1^ with the pyrophosphate of lipid II, which was observed previously by solid state NMR in hydrated membranes^10^ but not in the monomeric DMSO solution structure^23^. A recurring hydrogen bond is observed across all trajectories between the amine of Abu8 and the pyrophosphate oxygen in agreement with the DMSO structure^23^. The N-terminus of the second nisin interacts with the C-terminus of the pentapeptide of lipid II, specifically D-Ala5 (Fig. 7). This interaction is canonical in all trajectories. We propose that this interaction could be important in a preliminary recognition process that leads a second nisin to the binding site on lipid II and this could be tested *in vitro* using a laboratory synthesised lipid-II variant^16^, modified to have a methylated C-terminus on the pentapetide.

**Figure 8:**
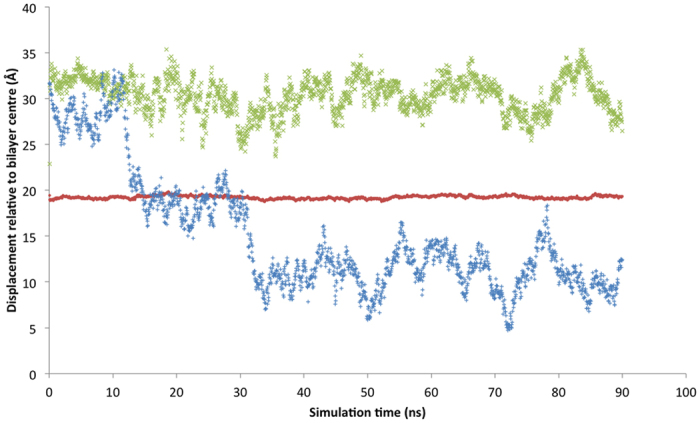
Example Z-direction displacement of centre of mass of (i) bilayer phosphates (red), (ii) nisin_1_ N-termini heavy atoms (green) and (iii) nisin_2_ N-termini heavy atoms (blue) for a trajectory run where nisin_2_ inserts into bilayer.

**Figure 9:**
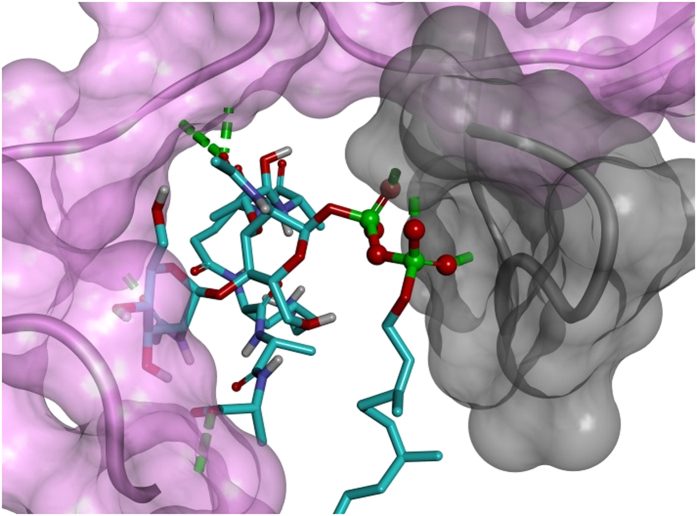
Coiled (**C**) conformation of the lipid II undecaprenyl chain in a membrane complex with nisin_2_. Nisin^1^ is shown as a grey ribbon and nisin^2^ as a pink ribbon. Membrane phosphates are shown to illustrate level of lipid head groups, other atoms removed for clarity.

During three of the six nisin_2_:lipid II trajectories the second docked nisin penetrates through the head group region of the membrane into the hydrophobic region. Figure 8 shows the z-direction displacement of the N-terminus of nisin^1^ and nisin^2^, and the level of the phosphate atoms in the lipids for reference, with respect to the bilayer centre, for an example of one of these trajectories. Figure 9 is an illustrative snapshot of the same trajectory, where the N-terminus of nisin^2^ is interacting with the C-terminus of the lipid-II pentapeptide. We propose that this interaction, observed across all six trajectories, allows both of these termini groups to insert into the membrane by reducing the energetic cost of locating a polar species in the hydrophobic bilayer region and is therefore the first step in pore formation.

## Conclusions

In this work we put forward a model of the ternary nisin_2_:lipid II complex, implicated as a key building block of oligomeric membrane lytic pores. We characterise a second, higher affinity binding site for a second nisin on the nisin:lipid II complex, which is essential for understanding the assembly of 8:4 nisin:lipid II pore complexes in bacterial membranes. The ternary nisin_2_-lipid II complexes were stable in six independent MD simulations, where the complex was embedded into bilayers of 3:1 POPG:POPE and fully hydrated. Additionally, the undecaprenyl chain of lipid II adopted a coiled conformation when lipid II formed a complex with two nisin molecules which is not seen with lipid-II alone. Hydrogen bond analysis reveals N-terminal engagement of both nisin molecules with lipid II, nisin^1^ to the pyrophosphate and nisin^2^ to the C-terminus of the pentapeptide chain. In half of the trajectories nisin^2^ is inserted into the membrane and we propose this is the first step in pore formation, mediated by the nisin^2^–pentapeptide hydrogen bond interaction. The results show good agreement with solid state NMR studies and extend solution NMR data from the complex. These findings support a proposed nisin_2_:lipid II stoichiometry and suggest that a second binding site on lipid II for nisin is located at the C-terminus of the D-Ala5 residue. At present, we are investigating a higher oligomeric state of the nisin:lipid II membranes complexes, built on the structure proposed here of the ternary complexes.

## Additional Information

**How to cite this article**: Mulholland, S. *et al.* Docking and molecular dynamics simulations of the ternary complex nisin_2_:lipid II. *Sci. Rep.* **6**, 21185; doi: 10.1038/srep21185 (2016).

## Supplementary Material

Supplementary Information

## Figures and Tables

**Figure 1:**
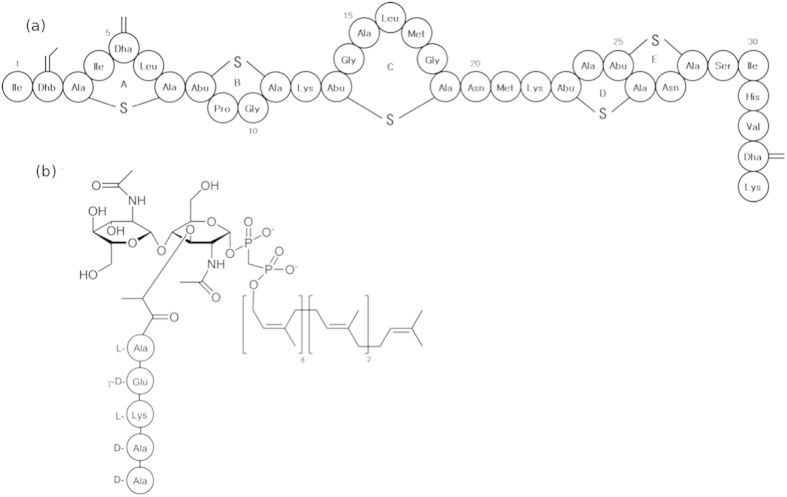
(**a**) Structure of wild-type nisin showing the lanthionine ring A, the β-methyl-lanthionine rings B, C, D and E, and the three dehydrated residues Dhb2, Dha5 and Dha33. (**b**) Chemical structure of lipid II, showing N-acetylmuramic acid and N-acetylglucosamine.

**Table 1:** Calculated binding affinity for nisin docking onto the three different models of lipid II.

Binding Affinity (kcal/mol)		
Model	Wild Type	lle1Trp
DMSO solvated	−11.0	−13.7
1-iso	−10.3	−12.3
3-iso	−11.7	−11.9

Ten torsional degrees of freedom in the nisin backbone were considered.

**Table 2:** Analysis of hydrogen bonding between polar moieties in nisin and the lipid II head group.

	Atom	Trajectory					
		1	2	3	4	5	6
Occupancy of interaction with pyrophosphate (%)							
Nisin 1	Ile1 HT1	5	22	23	0	0	0
	Ile1 HT2	4	25	25	0	0	0
	Ile1 HT3	4	22	24	0	0	0
	Dhb2 NH	4	2	17	0	0	0
	Ala3 NH	2	0	22	0	0	75
	Cys7 NH	2	1	22	7	8	0
	Abu8 NH	94	18	87	24	86	99
Nisin 2	Lys12 HZ1	0	0	0	37	30	60
	Lys12 HZ2	0	0	0	26	28	16
	Lys12 HZ3	0	0	0	28	26	15
	Asn20 HD21	0	19	0	0	0	0
	Lys22 NH	18	0	0	0	0	0
Occupancy of interaction with disaccharide (%)							
Nisin 2	Ile1 HT1	15	0	4	7	1	3
	Ile1 HT2	15	0	2	9	1	4
	Ile1 HT3	14	0	2	11	1	4
	Cys11 O	0	20	0	0	0	0
	Lys12 HZ1	0	42	0	0	0	3
	Lys12 HZ2	0	35	0	0	0	6
	Lys12 HZ3	0	29	0	0	0	3
	Asn20 HN	38	0	0	0	0	0
	Asn20 HD22	0	34	0	0	0	0
Occupancy of interaction with pentapeptide (%)							
Nisin 2	Ile1 HT1	34	64	50	24	30	7
	Ile1 HT2	29	37	59	48	27	6
	Ile1 HT3	30	69	49	28	29	10
	Dhb2 HN	1	16	25	93	0	0
	Asn20 OD1	0	25	0	0	0	0
	Asn20 HD21	16	0	0	0	0	0
	Met21 HN	75	0	0	0	0	0

Interactions are only shown where the occupancy was ≥15% across one of the trajectories. Atoms are defined by residue name, residue number and atom type. The occupancy is the percentage of trajectory frames where the hydrogen bond is formed.
