# Supplementary material for: Docking and molecular dynamics simulations of the ternary complex nisin2:lipid II
Source: Sci Rep. 2016 Feb 18;6:21185. doi: 10.1038/srep21185 (PMC4758073; doi:10.1038/srep21185)
Supplement: Supplementary Information [file srep21185-s1.pdf]

Supplementary Data

Docking and molecular dynamics simulations of the ternary complex nisin$_2$:lipid II

Sam Mulholland, Eleanor R. Turpin, Boyan B. Bonev*, Jonathan D. Hirst*

Docking of nisin to lipid I

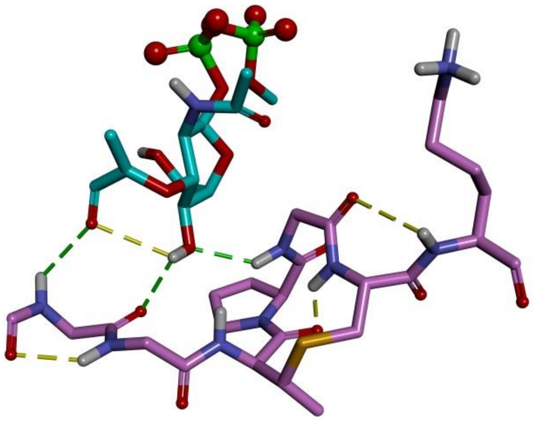

Figure S1 Docking of nisin^2 (pink carbon atoms) onto the complex formed between the first nisin (not shown) with lipid I (cyan carbon atoms) (groups on lipid I have been omitted for clarity). Intermolecular hydrogen bonding shown in green dashes, intramolecular hydrogen bonding in yellow. A hydrogen bond formed between Gly10 of nisin^2 and the O4 hydroxyl group on MurNAc, which also interacted with Leu6 of nisin^2 and the carbonyl in the scissile bond between the MurNAc and pentapeptide (intramolecular hydrogen bond depicted in yellow). The carbonyl on the MurNAc formed an additional hydrogen bond to Leu6 NH of nisin^2. There were multiple hydrophobic interactions from the peptide chain of lipid I to Leu6 and Pro9 of nisin and also from the isoprenyl chain to Lys12 in nisin^2.

MD simulation of Lipid II

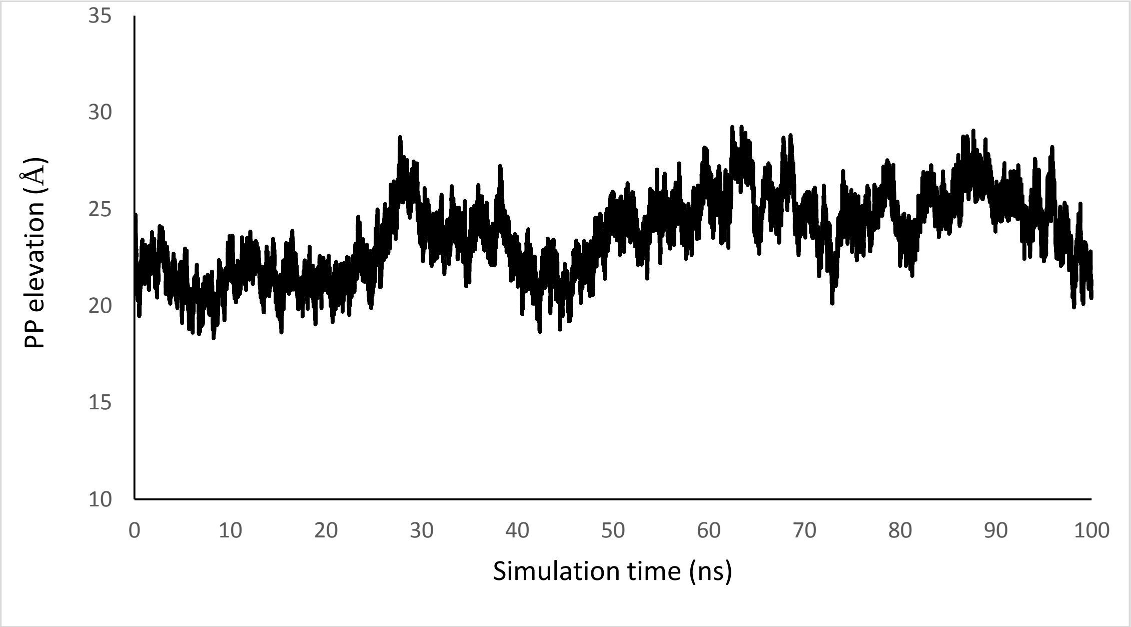

Figure S2 Elevation of pyrophosphate of lipid II relative to the centre of the lipid bilayer throughout the simulation.

Docking of nisin to MD models of lipid II

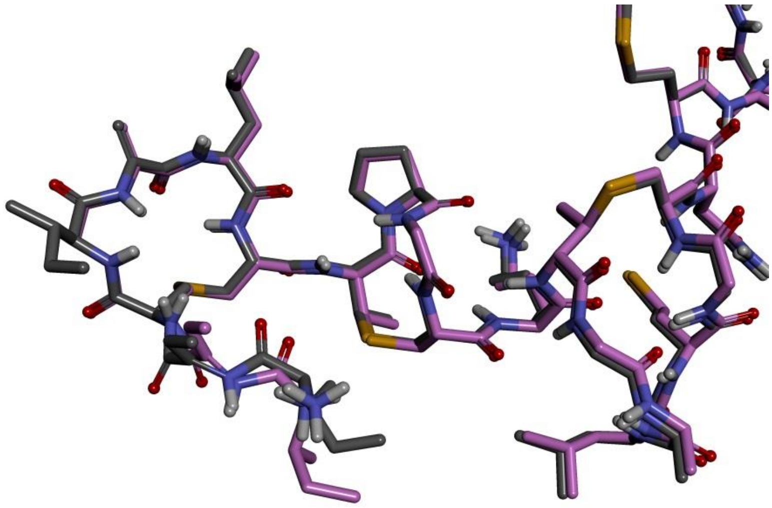

Figure S3 Two nisin conformations obtained after docking to the MD-derived model of lipid II with one isoprenyl unit (grey carbon atoms) and three isoprenyl units (pink carbon atoms).

MD Simulation of Nisin$_2$:lipid II

Å

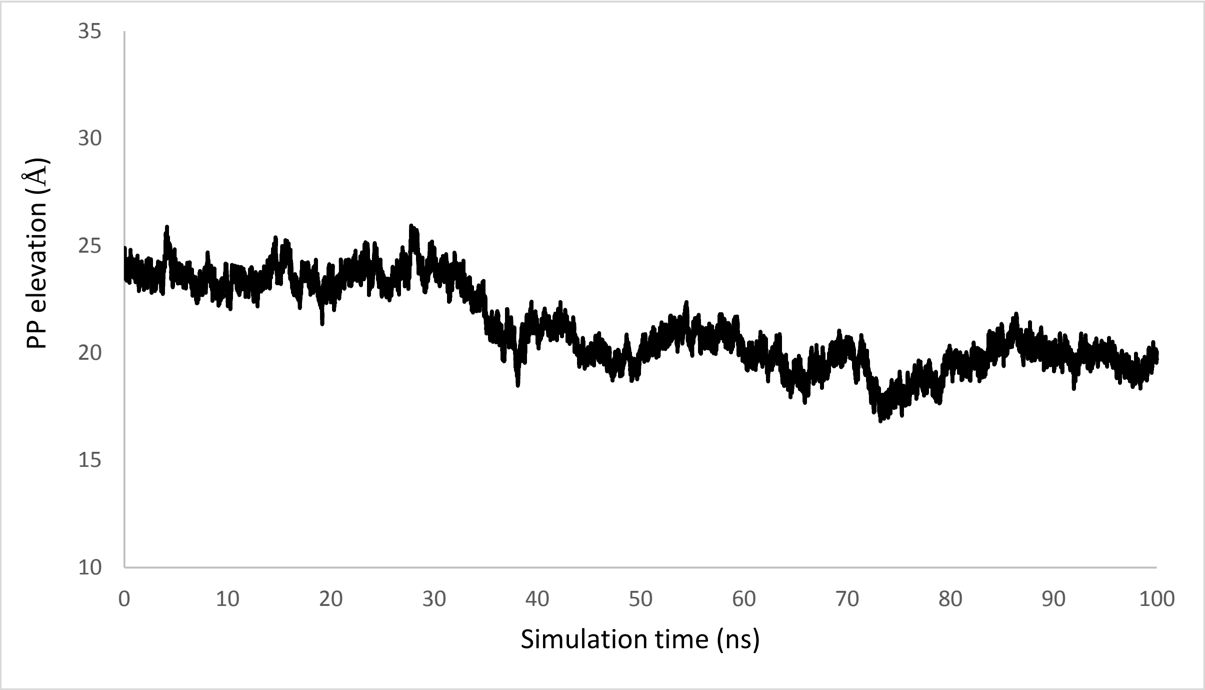

Figure S4 Elevation of the pyrophosphate in lipid II relative to the bilayer centre followed throughout the simulation with two nisin molecules bound to the complex.
